# GaN metal-organic vapor phase epitaxy on Sc_2_O_3_/Si templates for group III-nitride monolithic integration to Si technology

**DOI:** 10.1038/s41598-025-12904-9

**Published:** 2025-07-27

**Authors:** Tomas Grinys, Arūnas Kadys, Tadas Malinauskas, Petras Lapukas, Žydrūnas Podlipskas, Rimantas Gudaitis, Šarūnas Meškinis

**Affiliations:** 1 Institute of Photonics and Nanotechnology, Faculty of Physics, Vilnius University, 10257 Vilnius, Lithuania; 2 Institute of Material Science, Kaunas University of Technology, 51423 Kaunas, Lithuania

**Keywords:** Materials for devices, Materials science

## Abstract

In this work, we present a detailed analysis of GaN layers up to 500 nm thick, directly grown on Sc_2_O_3_(111)/Si(111) templates using metal-organic vapor phase epitaxy. A range of measurement techniques, including X-ray diffraction, Raman spectroscopy, atomic force microscopy, cathodoluminescence, and scanning electron microscopy (SEM), were used to evaluate structural quality, strain/stress states, surface morphology, and dislocation densities. The micro-stripe formation was observed when the growth was conducted in a nitrogen atmosphere, with the stripes completely disappearing when the growth atmosphere was switched to hydrogen. The stripes were determined to be of a cubic GaN phase. The epitaxial relationships between the cubic GaN crystalline lattice and Sc_2_O_3_, Si, and hexagonal GaN were examined in detail. Continuous, c-axis-oriented, monocrystalline GaN layers on Sc_2_O_3_ can be achieved in both $\hbox {N}_2$ and $\hbox {H}_2$ atmospheres. Prolonged nitridation processes of up to 1200 s improved the smoothness and crystallinity of the GaN layers, significantly reducing the number of extended defects. Switching the growth atmosphere from $\hbox {N}_2$ to $\hbox {H}_2$ led to reduced dislocation densities, minimized cubic GaN formation, and improved the surface morphology of the GaN layers. Our analysis shows that due to the lattice and thermal mismatch between GaN and the Si substrate, the GaN layers experience tensile strain. To manage this strain, $\hbox {Al}_x$ $\hbox {Ga}_{1-x}$N interlayers were inserted after 100 nm of GaN growth. This strain-engineering approach resulted in smooth, crack-free GaN epitaxial layers, demonstrating the potential for integrating GaN into silicon technology using a $\hbox {Sc}_{{2}}$ $\hbox {O}_{{3}}$.

## Introduction

III-nitride semiconductors, including GaN, AlN, and InN, are integral to advanced electronic devices, offering a broad spectrum of bandgap energies suitable for diverse applications. Silicon substrates are widely used for GaN film growth due to their cost-effectiveness and size scalability, yet they present challenges such as lattice and thermal expansion mismatches with III-nitrides. Technologists employ AlN, AlGaN, or superlattices with Al content as buffer layers on Si to mitigate these issues, which reduce defect propagation caused by lattice mismatches and address thermal expansion discrepancies^1,2^.

The direct epitaxy of GaN on silicon presents an opportunity to integrate photonic and electronic devices made from these different materials. Integrated photonic circuits consisting of light sources, photodetectors, and passive photonic elements such as waveguides can all be fabricated from the same III-nitride epitaxial layers grown on silicon^3–5^. The integration of circuits comprising GaN-based high-electron-mobility transistors (HEMTs) or light-emitting diodes (LEDs) with silicon complementary metal-oxide-semiconductor (CMOS) structures has also been demonstrated. While those integration technologies still focus on complicated flip-chip bonding techniques^6^, there are promising studies on direct selective growth on silicon via molecular beam epitaxy (MBE) or metal-organic vapor phase epitaxy for monolithic integration^7,8^.

Recent advances in the design of HEMT structures on silicon have enabled the development of integrated logic circuits consisting entirely of GaN^9,10^. Furthermore, the monolithic integration of both high- and low-power GaN electronics on a single substrate has gained increasing importance. Such a structure helps to avoid reduction of operating frequency and electrical noise caused by long wire interconnects between the device chip and low-power drivers. Silicon-on-insulator (SOI) wafers with SiO${_2}$ acting as insulator layers are commonly used for monolithic integration to minimize leakage current between separate devices^11,12^. Rare earth oxides, such as Er${_2}$O${_3}$ or Sc${_2}$O${_3}$, used as buffer layers, offer an alternative to SiO${_2}$ for monolithic integration. These single-crystalline rare earth oxides having a high dielectric constant^13,14^ allow for the direct epitaxy of GaN and offer the potential for achieving semi- and non-polar structures^15,16^. Due to the high refractive index contrast between silicon and rare-earth oxides, these materials are also promising for constructing Bragg mirrors, which can be used in photonic and optoelectronic applications^17,18^.

In this study, we report, for the first time, the epitaxy of GaN on Si substrates using a Sc${_2}$O${_3}$ buffer layer by the MOVPE technique. The lattice mismatch between Si(111) and GaN(0001) is approximately 17%^19^. However, introducing a buffer layer can reduce this significant difference, as the theoretical mismatch at room temperature between Sc${_2}$O${_3}$(111) and GaN(0001) is 8.4%^20^. While this research aims to determine the optimal conditions for hexagonal GaN epitaxy, the formation of a metastable cubic GaN phase can be expected^21–23^. Cubic GaN has a bandgap of 3.2 eV at room temperature, which is lower than that of the hexagonal phase 3.4eV^24,25^. The polarization effect observed in hexagonal GaN is absent in cubic GaN. This property of cubic GaN can be preferential for photonic device applications solving the efficiency problem, especially in the green wavelength range^26^. Consequently, a significant part of this study focuses on phase analysis of GaN formed during the MOVPE process.

## Results

### Lattice alignment

**Fig. 1:**
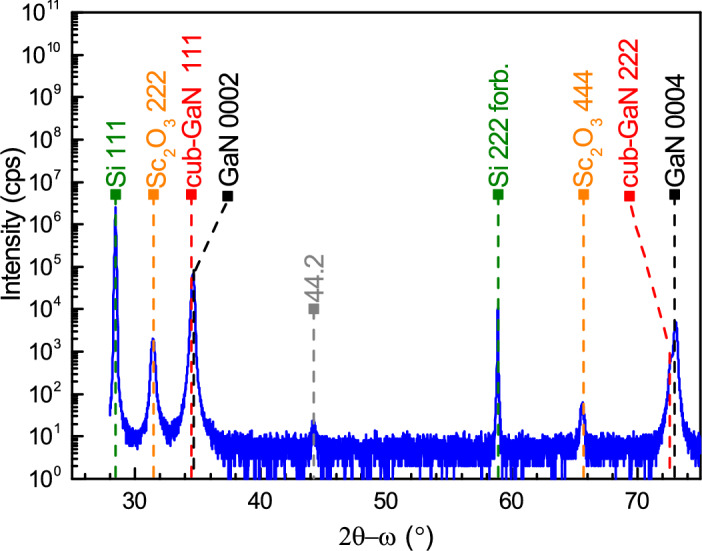
XRD patterns of GaN deposited on Si(111) with the $\hbox {Sc}_{{2}}$ $\hbox {O}_{{3}}$(111) interlayer grown under nitrogen atmosphere (sample No. 4).

A representative X-ray diffraction (XRD) spectrum in a broad 2$\theta -\omega$ range of GaN grown by MOVPE on a $\hbox {Sc}_{{2}}$ $\hbox {O}_{{3}}$(111)/Si(111) substrate is shown in Fig. 1. The dashed lines indicate the corresponding phases identified in the sample. The dominating GaN phase corresponds to a c-plane, single-crystalline wurtzite structure. Both the 0002 and 0004 reflections of hexagonal GaN are shifted towards higher diffraction $2\theta$ angles compared to their theoretical relaxed values. This shift to higher angles is associated with tensile in-plane strain, leading to an increase of the in-plane lattice constant $\hbox {a}_{{GaN}}$ and a reduction of out-of-plane lattice constant $\hbox {c}_{{GaN}}$. Upon further analysis of the GaN 0002 and 0004 peaks, a shoulder at a lower diffraction angle is noticeable. This shoulder corresponds to 111 reflection of an additional cubic GaN (cub-GaN) phase formed during epitaxial growth (see also Fig. 3b for spectra in a lower 2$\theta -\omega$ range). Moreover, a low-intensity peak was detected at 44.2$^\circ$, which can be attributed to the $\hbox {Sc}_{{2}}$ $\hbox {O}_{{3}}$(111)/Si(111) template. This peak is likely due to the formation of scandium oxide silicides ($\hbox {Sc}_x$ $\hbox {Si}_{1-x}$ $\hbox {O}_y$) or silicate ($\hbox {SiO}_x$) at the interface during the MBE epitaxy process^27–29^.

**Fig. 2:**
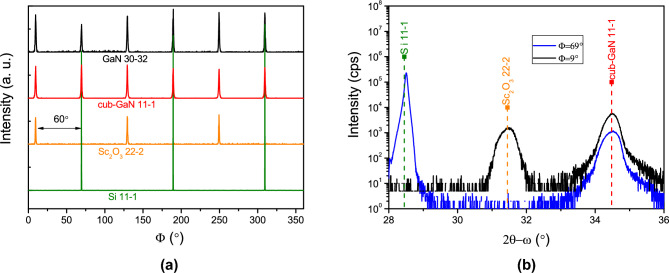
XRD patterns of sample No. 4. (**a**) $\Phi$ scan of GaN deposited on Si(111) with the $\hbox {Sc}_{{2}}$ $\hbox {O}_{{3}}$(111) interlayer grown under nitrogen atmosphere: across Si$\{$11-1$\}$, $\hbox {Sc}_{{2}}$ $\hbox {O}_{{3}}$ $\{$22-2$\}$, cub-GaN$\{$11-1$\}$, GaN$\{$30-32$\}$ lattice planes. (**b**) Skew symmetric scan 2$\theta -\omega$ scan at $\chi =70.5^\circ$ of GaN on Si with the $\hbox {Sc}_{{2}}$ $\hbox {O}_{{3}}$ interlayer grown in nitrogen atmosphere: aligned to Si 11-1 reflection $\Phi =69^\circ$, and $\hbox {Sc}_{{2}}$ $\hbox {O}_{{3}}$ 22-2 crystalline twin at $\Phi =9^\circ$. The intensity of the X-ray diffraction spectrum at $\Phi =9^\circ$ was multiplied by 5 for a better view.

The azimuthal orientation of all phase components identified in the grown GaN(0001) on $\hbox {Sc}_{{2}}$ $\hbox {O}_{{3}}$(111)/Si(111) sample is presented in Fig. 2a . The X-ray diffraction $\Phi$ scan reveals a three-fold symmetry for the Si$\{$11-1$\}$ and $\hbox {Sc}_{{2}}$ $\hbox {O}_{{3}}$ $\{$22-2$\}$ reflections, while a six-fold symmetry is observed for the cubic GaN$\{$11-1$\}$ and hexagonal GaN$\{$30-32$\}$ families of lattice planes. The six-fold symmetry of GaN confirms its wurtzite lattice structure. However, the exact six-fold symmetry observed for cubic GaN indicates the presence of two in-plane twins rotated by a 60$^\circ$ angle. This result was also observed for GaN grown via MBE on $\hbox {Sc}_{{2}}$ $\hbox {O}_{{3}}$ (111)/$\hbox {Y}_{{2}}$ $\hbox {O}_{{3}}$ (111)/Si(111) substrate^29^. Additionally, it is worth noting that the twin-free $\hbox {Sc}_{{2}}$ $\hbox {O}_{{3}}$ $\{$22-2$\}$ lattice planes are rotated by 60$^\circ$ relative to the Si$\{$11-1$\}$ planes. This configuration reveals an AB stacking relationship, typical for single-crystalline cubic bixbyite oxide structures grown epitaxially on Si(111). The AB stacking arrangement is energetically more favorable than the AA configuration^28,30^.

The X-ray diffraction measurements were carried out in a skew-symmetric geometry to confirm the formation of the cubic GaN(111) phase during growth and to distinguish it from the nearby hexagonal GaN 0002 peak. By tilting the diffraction plane at an angle of $\chi =70.5^\circ$, only the $\{$11-1$\}$ planes of the cubic lattice cell were analyzed, effectively excluding any diffraction from the hexagonal structure’s atomic planes. Figure 2b presents the X-ray diffraction spectra measured at two different azimuthal orientations: $\Phi$ = 9$^\circ$ and $\Phi$ = 69$^\circ$. A strong cubic GaN 11-1 peak is visible in both azimuthal orientations, confirming the presence of twinning. The azimuthal relationship between cubic GaN and $\hbox {Sc}_{{2}}$ $\hbox {O}_{{3}}$ can be expressed as $\hbox {Sc}_{{2}}$ $\hbox {O}_{{3}}$[-1-12]$\Vert$cub-GaN[11-2] or $\hbox {Sc}_{{2}}$ $\hbox {O}_{{3}}$[-1-12]$\Vert$cub-GaN[-1-12], corresponding to the respective twin orientations.

**Fig. 3:**
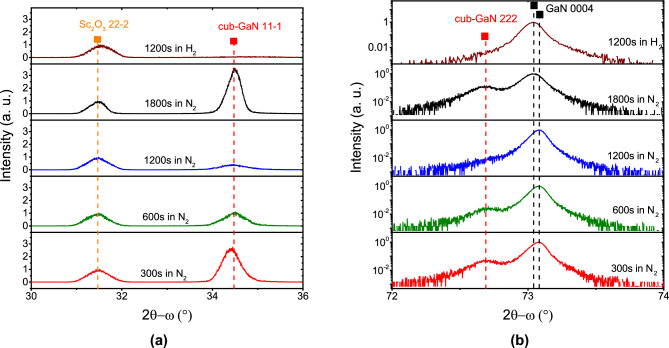
XRD patterns of samples No. 1–4 and No. 8. (**a**) Skew symmetric 2$\theta -\omega$ reflections of GaN layers grown at different nitridation time in $\hbox {N}_{{2}}$ and $\hbox {H}_{{2}}$ atmosphere ($\chi =70.5^\circ$, and $\Phi =9^\circ$). The reflection intensity was normalized to $\hbox {Sc}_{{2}}$ $\hbox {O}_{{3}}$ 222 peak. (**b**) Symmetric $2\theta -\omega$ reflections of GaN layers grown at different nitridation time in $\hbox {N}_{{2}}$ and $\hbox {H}_{{2}}$ atmosphere. The reflection intensity was normalized to the hexagonal GaN 0004 peak.

Figure 3a shows the XRD skew-symmetric scan reflections of GaN layers grown with varying nitridation times. All XRD reflections were normalized to the $\hbox {Sc}_{{2}}$ $\hbox {O}_{{3}}$ 222 peak to allow for a direct comparison of the nitridation effect. The intensity of the peak at 2$\theta =34.45^\circ$ corresponding to the amount of cubic GaN phase does not remain constant with varying nitridation time. There are optimal conditions at a nitridation time of 1200 s to get the lowest amount of cubic GaN phase in N${_2}$ atmosphere. Notably, only traces of cubic GaN remained when the growth atmosphere was changed from N${_2}$ to H${_2}$, suggesting that hydrogen is responsible for the dissociation of the cub-GaN phase.

### Effect of nitridation time and growth atmosphere

Figure 3b shows the XRD symmetric 2$\theta -\omega$ scan normalized to the 0004 peak of hexagonal GaN. Those 0004 reflectance measurements at a higher 2$\theta$ angle allow better separation between the hexagonal and cubic GaN phases compared to 0002 reflectance. The shoulder peak corresponding to the cubic GaN phase follows the tendency of that observed in the skew-symmetric scan (Fig. 3a), confirming that the nitridation time influences the amount of cubic GaN. Furthermore, the nitridation time affects the position of the hexagonal GaN 0004 peak. The position remains almost unchanged up to 1200 s in an $\hbox {N}_2$ atmosphere, while the GaN peak shifts to a lower 2$\theta$ angle for longer nitridation time (1800 s) or when the growth atmosphere is changed from $\hbox {N}_2$ to $\hbox {H}_2$. This shift to a lower angle, theoretical relaxed value of hexagonal GaN reveals the lower tensile strain state of the GaN. Such results suggest that the inclusions of the cubic GaN phase affect the strain state of hexagonal GaN.

### Surface morphology

**Fig. 4:**
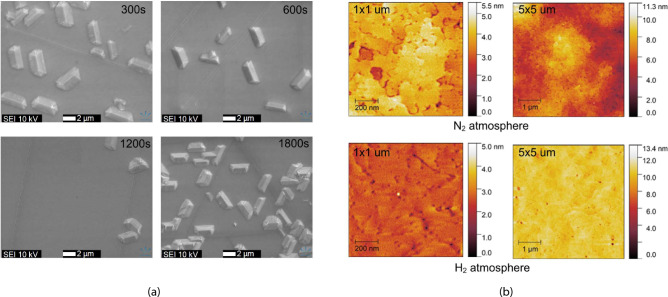
Change of surface morphology. (**a**) with nitridation time 300 s, 600 s, 1200 s, 1800 s (SEM images of samples No. 1–4). (**b**) GaN grown in nitrogen and hydrogen atmosphere, respectively (AFM images of samples No. 3 and No. 8).

The surface morphology analysis of the GaN layer grown in $\hbox {N}_{{2}}$ atmosphere at different nitridation times is shown in Fig. 4a . Straight dark lines corresponding to particular crystallographic orientations were observed in the SEM image, which are identified as cracks. These cracks form in the GaN layer during cool down from growth temperature to room temperature, caused by the mismatch in thermal expansion coefficients between GaN and Si. The crack formation was also observed in GaN layers grown in the $\hbox {H}_{{2}}$ atmosphere. In addition to cracks, stripe-type defects form on the surface of GaN layers grown in the $\hbox {N}_{{2}}$ atmosphere. The cross-section of the stripes towards the longer side direction shows a triangular shape bound by two facets. The percentage of the surface area covered by stripes closely correlates with the amount of cubic GaN. A higher amount of cubic GaN formed during the nitridation process leads to a higher percentage of the area covered by stripes. Changing the growth atmosphere from $\hbox {N}_{{2}}$ to $\hbox {H}_{{2}}$ results in the disappearance of stripes and a smoother surface (see Fig. 8). It should be noted that although the percentage of the area covered by stripes is similar for both the shortest (300 s) and longest (1800 s) nitridation times, the larger stripes were observed at shorter nitridation times, while smaller but denser stripes were observed for the longest nitridation time.

The representative AFM analysis in the areas free of stripe-type defects of GaN surface grown in nitrogen and hydrogen atmospheres are shown in Fig. 4b . The surface roughness is lower for the samples grown in a hydrogen atmosphere. The root mean square (RMS) of $1\times 1$ $\upmu$ $\hbox {m}^{2}$ is 0.3 nm for the GaN layer grown in a hydrogen atmosphere and 0.5 nm in a nitrogen atmosphere. For larger $5\times 5$ $\upmu$ $\hbox {m}^{2}$ area scans, the roughness increases to 0.5 nm and 1.5 nm in hydrogen and nitrogen atmosphere, respectively. Such values indicate a smooth surface typical for GaN on Si technology^2,31^. Hydrogen takes place in the main GaN formation chemical reaction: Ga+$\hbox {NH}_{3}\rightarrow$GaN+3/$\hbox {2H}_{{2}}$. As a result, it shifts the reaction equilibrium to the left, promoting the enhanced dissociation of GaN. The dissociation of defective sites is preferred due to their higher surface energy and reduced stability compared to the surrounding hexagonal GaN^32,33^. Therefore, lower roughness can be expected for GaN growth in a hydrogen atmosphere. Shallow pits can be clearly distinguished in the $1\times 1$ $\upmu$ $\hbox {m}^{2}$ AFM image of the GaN layer grown in a hydrogen atmosphere (Fig. 4b). These pits correspond to the open core of screw, edge, and mixed types of threading dislocations^34^. The density of pits is $1.5\cdot 10^{10}$ $\hbox {cm}^{-2}$, determined from AFM image analysis. This value is almost an order of magnitude higher compared to the $3.6\cdot 10^{9}$ $\hbox {cm}^{-2}$ of screw-type threading dislocations determined from XRD measurements for this sample. Usually, around 10 % of overall threading dislocations belong to the screw, mixed component^35,36^.

### Effect of thickness

**Fig. 5:**
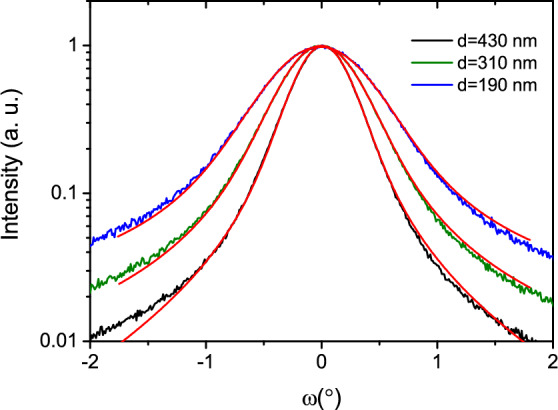
Rocking curve scan of GaN 0002 reflection (samples No. 5–7). Blue, green and black curves shows experimental data, while red curve presents fitting to Voigt function.

High-resolution XRD analysis of GaN layers of varying thicknesses grown in a nitrogen atmosphere is shown in Fig. 5. The XRD rocking curve measurements of reflections from the GaN 0002 lattice plane revealed a full width at half maximum (FWHM) of 1.0$^\circ$, 0.8$^\circ$, and 0.5$^\circ$ for samples with thicknesses of 190 nm, 310 nm, and 430 nm, respectively. According to the model proposed by Dunn and Koch^37^, the density of screw-type threading dislocations can be calculated using the equation $\varrho =\beta ^2/(4.36b^2)$, where $\beta$ is the peak broadening and *b* is the magnitude of the Burgers vector. This model gives screw, mixed-type threading dislocation density values of $\varrho =$ 2.6 $\times 10^{10}$, 1.5 $\times 10^{10}$, and 8.2 $\times 10^{9}$ $\hbox {cm}^{-2}$ for the samples of corresponding thicknesses. The reduction of such extended defects with increasing thickness is typical for epitaxial crystalline layer growth. Mechanisms such as fusion or annihilation reactions of dislocations are usually responsible for the decrease in their number^35,38^.

### Cathodoluminescence

**Fig. 6:**
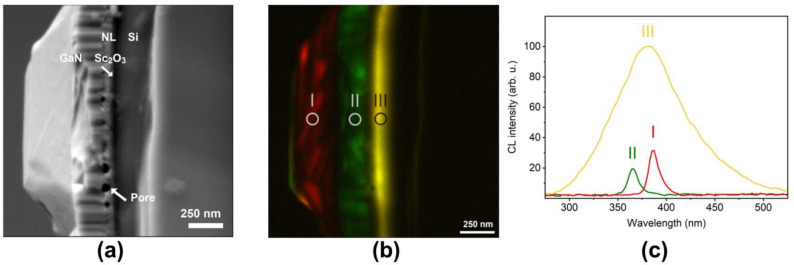
Cathodoluminescence measurements: (**a**) SEM cross-sectional image of GaN grown on Si with a Sc${_2}$O${_3}$ buffer layer, (**b**) corresponding CL image, and (**c**) CL spectrum. The sample was grown in a nitrogen atmosphere with a nitridation time of 1800 s. Label NL corresponds to GaN nucleation layer in SEM image.

To investigate the cathodoluminescence (CL) properties, sample No. 4 was cleaved to expose its cross-section. Figure 6 shows the layer structure at the cut location, along with a side view of a GaN micro-stripe. In this bird’s-eye cross-sectional view, the height of the protruding GaN micro-stripe is approximately 350 nm, while the total thickness of the GaN layer is around 270 nm, as determined from the SEM image in Fig. 6a. A distinct low-temperature GaN nucleation layer, approximately 50 nm thick, can be identified, indicating that the high-temperature GaN layer above it has a thickness of roughly 220 nm. Energy-dispersive X-ray (EDX) analysis (see supplementary material Fig. S1) confirmed that the dark region observed in the SEM image, approximately 40 nm thick and located just below the nucleation layer, corresponds to the Sc${_2}$O${_3}$ buffer layer. This particular sample was grown in a nitrogen atmosphere with the highest nitridation time of 1800 s. Such a long nitridation duration is responsible for the formation of pores in the GaN nucleation layer. This sample exhibits the highest density of micro-stripe structures, making it the most suitable sample for studying luminescence properties by CL.

The total emission spectrum from this sample consists of three distinct peaks (Fig. 6c). The peak at approximately 366 nm (3.39 eV) corresponds to a tensile-strained hexagonal GaN phase, whereas the peak at 386 nm (3.21 eV) is attributed to the cubic GaN phase. As one can see in Fig. 6b, the band-edge emission of the hexagonal GaN phase originates from the flat zones of the GaN layer, whereas the luminescence of the cubic phase arises from the micro-stripe structures. This suggests that the micro-stripe structures formed in a nitrogen atmosphere are inclusions consisting of cubic lattice cell within the hexagonal gallium nitride matrix.

It is also notable that intense luminescence was observed below the MOVPE-grown GaN layer within the Sc${_2}$O${_3}$ buffer. The band gap of Sc${_2}$O${_3}$ is 6.3 eV^39^. This broad luminescence, centered at 379 nm (3.27 eV), corresponds to the optical transitions within the band gap of Sc${_2}$O${_3}$ and can be related to defects such as oxygen vacancies (F-type centers)^40–42^, in this MBE-grown film.

### GaN with AlGaN interlayers for strain compensation

**Fig. 7:**
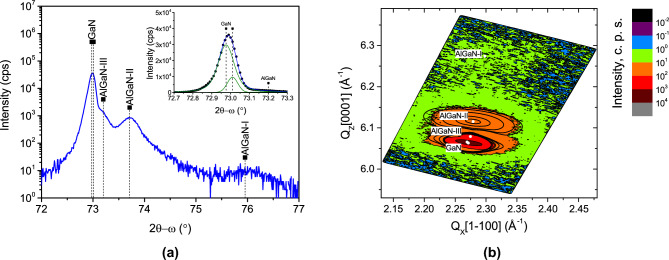
XRD measurements of GaN (sample No. 11) with AlGaN interlayers deposited in $\hbox {H}_{{2}}$ atmosphere on $\hbox {Sc}_{{2}}$ $\hbox {O}_{{3}}$(111)/Si(111). Symmetric 2$\theta -\omega$ scan (**a**). Reciprocal space map acquired around 10–15 reflection (**b**). Solid ellipsoidal curves represent the 2D Voigt function fitting. White points represent the maximum intensity positions obtained after fitting. White point of AlGaN-I layer added for the eye guide.

Additional AlN and AlGaN interlayers can be inserted to compensate for strain and prevent crack formation during GaN growth on silicon^1^. Three AlGaN layers with different Al content were inserted after the growth of a 130 nm optimized GaN buffer layer on Sc${_2}$O${_3}$. The final 440 nm GaN grown on the top of AlGaN interlayers was crack-free. The high-resolution $2\theta -\omega$ XRD spectrum on a semi-log scale for the same GaN sample with AlGaN interlayers is shown in Fig. 7a . Two separate peaks at $2\theta = 75.9^\circ$ and $73.7^\circ$, along with a shoulder at $73.2^\circ$, can be distinguished, corresponding to three AlGaN phases labeled as AlGaN-I, AlGaN-II, and AlGaN-III. To evaluate the strain state and aluminum composition of the interlayers, a reciprocal space map (RSM) around the 10$\bar{1}$5 reflection was measured, as shown in Fig. 7b . The weak diffraction peak labeled AlGaN-I corresponds to the first AlGaN layer, which has the highest Al content. Due to its nominal thickness of only 30 nm, the position of this layer can not be clearly determined in the RSM. However, the exact position of the AlGaN-II peak is clearly seen in the RSM, and fitting the data using a 2D Voigt function enabled the determination of its lattice parameters. Based on this analysis, the aluminum content and in-plane strain of the AlGaN-II layer were found to be x = 0.20 and $\varepsilon _{xx}=0.31\%$, respectively. The final AlGaN-III layer contains the lowest aluminum concentration, and its diffraction peak appears very close to that of GaN. After deconvolution of the RSM data, the aluminum content of this layer was estimated to be x = 0.06, with an in-plane strain of $\varepsilon _{xx}=0.21\%$.

**Fig. 8:**
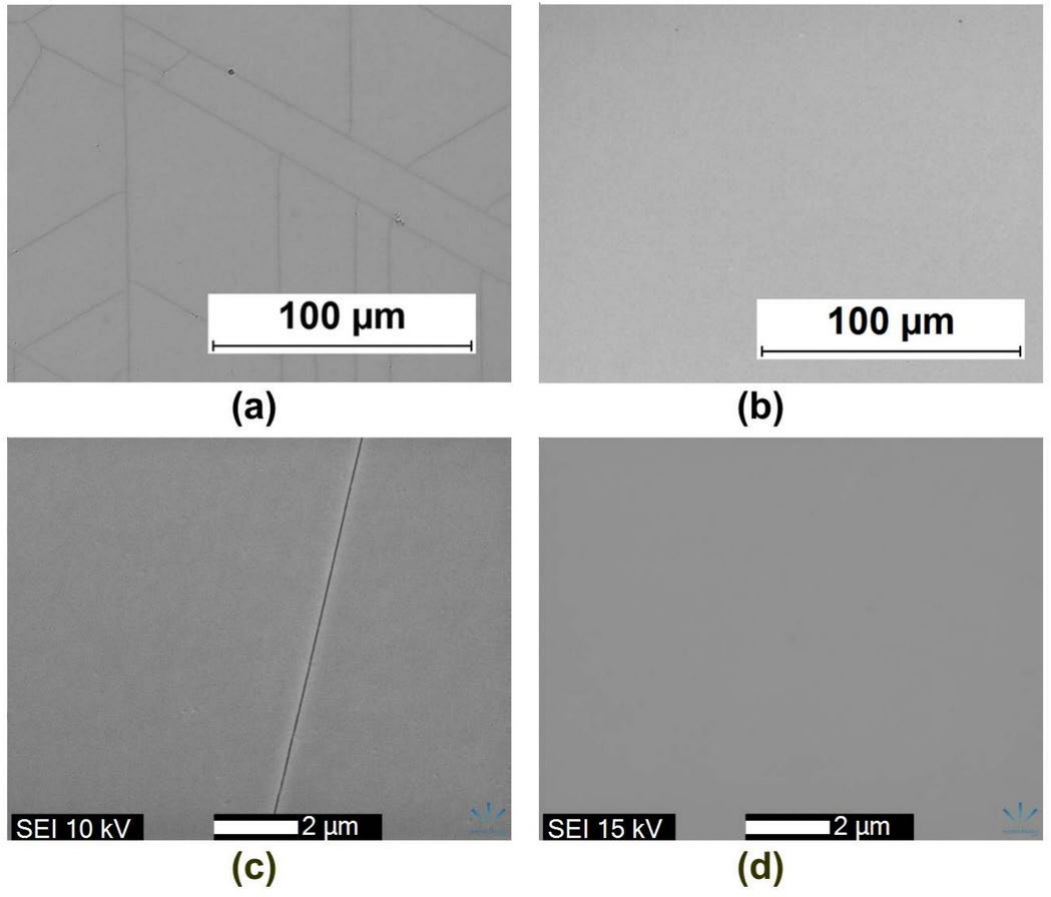
Optical microscope and SEM images of surface morphology (samples No. 8 and No. 11): without (**a**), (**c**) and with (**b**), (**d**) AlGaN interlayers.

An enlarged linear-scale view around the GaN 0004 peak is provided in the inset of the Fig. 7a . The GaN peak at approximately $2\theta = 73^\circ$, depicted on a linear scale, is asymmetric. This differs from the highly symmetric peaks observed without AlGaN interlayers, as described in previous sections. The asymmetric peak can be deconvoluted into two peaks at different $2\theta$ angles, revealing two strain states of GaN. The peak with the lower area corresponds to GaN in a high in-plane tensile strain state, with $\varepsilon _{xx} = 0.25\%$. This residual strain is similar to that of GaN ($0.32\%$) grown in an $\hbox {H}_2$ atmosphere without AlGaN interlayers, suggesting it can be attributed to the GaN layer grown directly on $\hbox {Sc}_2$ $\hbox {O}_3$. The peak with the much higher area corresponds to GaN in a low tensile strain state ($\varepsilon _{xx} = 0.16\%$). This layer can be attributed to the top 440 nm GaN layer grown on the AlGaN interlayers. These two GaN stress states were indicated in the reciprocal space map by two adjacent points (see Fig. 7b). The observed situation in the GaN layers is similar to previous work^36^, where two distinct GaN strain states were observed in epitaxial films separated by an AlN layer. The presence of two different remnant strain states in GaN and the reduced tensile strain in the upper GaN layer demonstrate the effectiveness of the AlGaN interlayers as strain-compensating functional layers. A comparison of the surface morphology of GaN layers grown without and with AlGaN interlayers in an $\hbox {H}_2$ atmosphere is presented in Fig. 8. Crack formation was suppressed by additionally inserting AlGaN layers.

### Raman measurement analysis

**Fig. 9:**
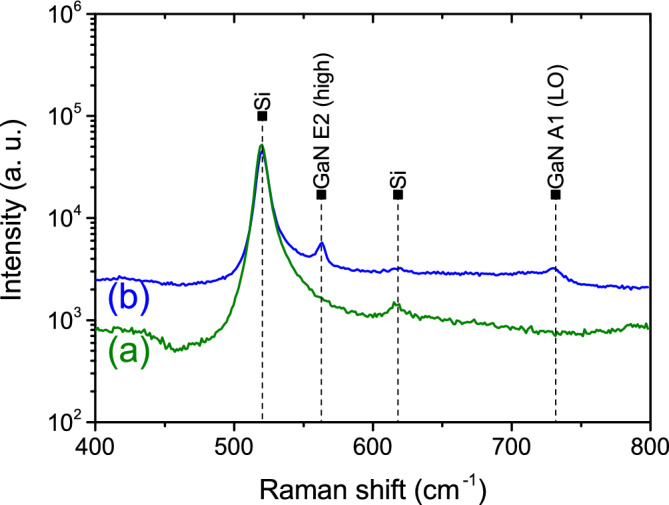
Typical Raman spectrum of $\hbox {Sc}_{{2}}$ $\hbox {O}_{{3}}$(111)/Si(111) before (**a**) and after (**b**) GaN growth in hydrogen atmosphere (sample No. 8).

The typical Raman spectrum of the $\hbox {Sc}_{{2}}$ $\hbox {O}_{{3}}$(111)/Si(111) substrate before GaN growth is shown in Fig. 9a. The strong peak at 520.3 $\hbox {cm}^{-1}$ and the weak one at 618 $\hbox {cm}^{-1}$ belong to the Si^43^. Two additional peaks of GaN E2 (high) and A1 (LO) vibrational modes^44^ at 564.8 $\hbox {cm}^{-1}$ and 731.7 $\hbox {cm}^{-1}$ appear after GaN growth in hydrogen atmosphere Fig. 9b. The change in Raman shift value $\Delta \varpi$ of the GaN peak E2 (high) is proportional to the remnant biaxial stress of GaN layer $\Delta \varpi =K\sigma$, were coefficient K is equal to 4.3 $\hbox {cm}^{-1}$ $\hbox {GPa}^{-1}$^31,45,46^, and $\sigma$ is the stress value of the GaN layer. For the completely relaxed GaN E2 (high) position is at 568 $\hbox {cm}^{-1}$, therefore the change to a lower Raman shift value gives the tensile stress equal to 1.2 GPa.

**Fig. 10:**
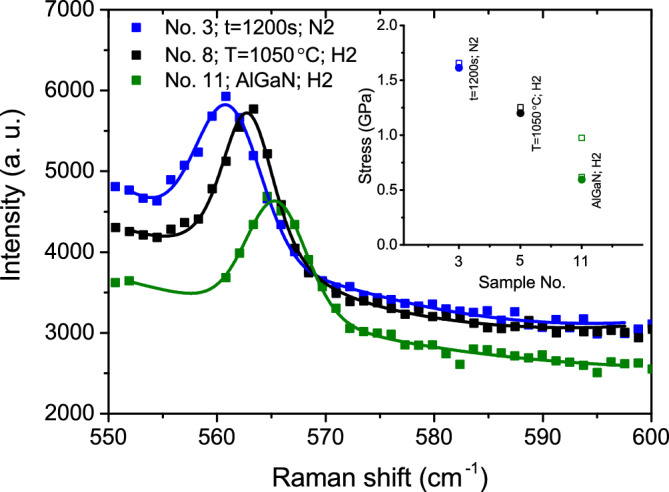
Comparison of GaN E2 (high) Raman spectra. Dots represent measured experimental values, while solid curves depict fitting to the Voigt function, along with a third-order polynomial for the background. The inset shows calculated stress values from XRD (open dots) and Raman (filled dots) measurements for the corresponding samples.

We compared the Raman peaks at the E2 (high) phonon mode of the sample grown in a nitrogen atmosphere with those of samples grown in a hydrogen atmosphere, including one with AlGaN interlayers. All three representative samples with the same nitridation time of 1200 s show different Raman peak positions (see Fig. 10). The Raman shift peak of the sample grown in $\hbox {N}_2$ atmosphere shows the highest displacement from the GaN stress-free position, while the sample with additional AlGaN interlayers shows the lowest displacement, indicating the lowest tensile stress of 0.6 GPa in the GaN layer. According to the relationship $\sigma =E\varepsilon _{xx}/(1-\nu )$^47^ between in-plane stress and strain, stress was also evaluated from previous strain values of the corresponding samples determined by XRD. In this relationship, the constant *E* (Young’s modulus) was chosen to be 324 GPa^48^. The calculated stress values (filled dots) from the Raman shift, as well as the stress values (open square dots) extracted from XRD measurements, are given in the inset of Fig. 10. Both measurements show close results, indicating that all GaN layers grown on $\hbox {Sc}_{{2}}$ $\hbox {O}_{{3}}$(111)/Si(111) are under tensile stress. The inserted AlGaN layer greatly reduces stress, leading to a crack-free GaN layer. It should be noted that, contrary to XRD measurements, only a single strain state of GaN was possible to determine from Raman measurements due to lower resolution of this method.

## Discussion

**Fig. 11:**
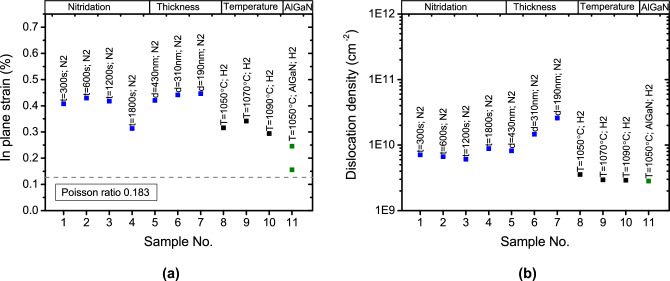
Comparison of in plane strain (**a**) and dislocation density (**b**) for all samples under investigation. Gray dotted line represents calculated value of thermal tensile strain induced during cooldown to room temperature.

The majority of GaN samples on $\hbox {Sc}_{{2}}$ $\hbox {O}_{{3}}$(111)/Si(111) was grown at a temperature T=1050$^\circ$C. Due to the higher thermal coefficient of GaN compared to the Si substrate, the tensile strain is induced in the GaN layer when cooling to room temperature. The induced in-plane strain $\Delta \varepsilon _{xx}$ can be evaluated by the following equation^49^:

1 $$\begin{aligned} \Delta \varepsilon _{xx}=\int (\alpha _{GaN}(T)-\alpha _{Si}(T))dT \end{aligned}$$

where $\alpha _{GaN}(T)$ and $\alpha _{Si}(T)$ are the coefficients of thermal expansion for the GaN layer and Si substrate material. Integrating the equation above from growth to room temperature and using data found in literature^50–52^ the $\Delta \varepsilon _{xx}$ was evaluated to be 0.13%. This value as a dashed line is presented in Fig. 11a. The in-plane strain values of all samples used in this research determined from XRD $2\theta -\omega$ scan are above this line, indicating that GaN layers are under tensile strain at the growth temperature.

Figure 11b shows the comparison of dislocation density for the samples under study, as determined from the XRD 0002 rocking curve. Samples No. 5, 6, and 7 in Fig. 11 were grown under the same conditions, with a growth time resulting in different thicknesses being the only variable parameter. We observed that the dislocation density decreases as thickness increases. As shown in Fig. 11a , GaN tends to relax with increasing thickness. Generally, the generation or annihilation of dislocations is related to changes in strain within the layer during epitaxy. At the beginning of the process, below a certain critical epitaxial layer thickness, the film can grow pseudomorphically. Plastic relaxation through the formation of dislocations occurs when this critical thickness is reached, which is directly proportional to the lattice mismatch between the substrate and the epitaxial layer. In our case, the lattice mismatch between GaN(0001) and $\hbox {Sc}_{{2}}$ $\hbox {O}_{{3}}$(111) is still significant, exceeding 8%. Partial relaxation and the generation of defects, such as misfit dislocations, may occur at the nanometer critical thickness scale. The subsequent change in dislocation density observed in samples No. 5, 6, and 7 is related to different mechanisms responsible for the bending and interaction of dislocations, which lead to a reduction in their number as the thickness increases.

Two groups of GaN samples can be distinguished in Fig. 11 based on their strain state and the number of dislocations. The first group consists of samples No. 1–7, exhibiting an in-plane tensile strain higher than 0.4%. A particular case is sample No. 4, which has a lower tensile strain value (see also a plot of dislocation density vs. in-plane strain Fig. S2 in supplementary for explanation). This sample was grown with a very high nitridation time, leading to structural degradation (appearance of pores) in the $\hbox {Sc}_{{2}}$ $\hbox {O}_{{3}}$ buffer layer, as well as the formation of a significant amount of cubic GaN. The degradation of the buffer layer may be responsible for the relaxation observed. All the samples in the first group were grown in an $\hbox {N}_2$ atmosphere.

The second group of samples, No. 8–11, were grown in an $\hbox {H}_2$ atmosphere and were more relaxed than the first group. Furthermore, the second group contains fewer dislocations than the first group. Therefore, summarizing the growth results, we can conclude that introducing $\hbox {H}_2$ promotes relaxation and reduces the dislocation density in the GaN layer.

Based on these collective results and recalling the effect of nitridation on the structural and morphological properties of GaN films, we propose an explanation for GaN micro-stripe formation. During the nitridation process, the ammonia molecules adsorb on the surface. The dissociated $\hbox {NH}_3$ molecules are the source of nitrogen and hydrogen atoms. By analogy with the nitridation process on sapphire substrates^53^, adsorbed nitrogen can form direct bonds with Sc-O or diffuse into the Sc${_2}$O${_3}$ layer^54^, replacing oxygen atoms. The out-diffused oxygen can then react with hydrogen at the surface, forming H${_2}$O vapor. As a result, we can expect that structures of compounds such as $\hbox {Sc}_{{x}}$ $\hbox {O}_{{y}}$ $\hbox {N}_{{z}}$ or even ScN will be formed. Given its threefold crystal symmetry and negligible lattice mismatch with GaN (less than 0.1%), ScN is expected to be preferential for cubic GaN growth. Sc-N bonds can be affected and dissociated by hydrogen atoms unless crystalline grains of critical size resistant to hydrogen are formed. The competition between ScN formation and dissociation reaches an extremum at around 1200 s, corresponding to the lowest density of nucleation sites for metastable cubic GaN growth.

Cuic GaN micro-stripes form during the initial growth phase in a nitrogen atmosphere (see supplementary material Fig. S3) and become embedded into the hexagonal GaN matrix at the final growth stage. The low-temperature GaN nucleation layer initially follows the three-dimensional Volmer-Weber growth mode^55^. Tensile strain is induced during the coalescence stage, where individual nuclei merge to form a continuous layer^56,57^. This strain generation is associated with the reduction of surface energy during layer formation^58^. Since the surface energy of metastable cubic GaN is higher than that of the hexagonal phase, the presence of cubic GaN in samples grown in an $\hbox {N}_{{2}}$ atmosphere results in higher residual tensile strain at the final growth stage compared to those grown in $\hbox {H}_{{2}}$, where only traces of the cubic phase remain. Furthermore, the interface between cubic and hexagonal GaN phases can act as a source of extended defects, contributing to a higher dislocation density in samples grown in $\hbox {N}_{{2}}$ atmosphere, as shown in Fig. 11b .

Moram et al. demonstrated that epitaxial crystalline ScN(111) typically exhibits a rough surface morphology. Such surface roughness leads to a reduced density of nucleation centers, resulting in pore formation, as shown in their study^59^. Based on their results and our previous discussion, we expect that at high nitridation times, a non-continuous crystalline ScN layer thicker than a monolayer forms on the Sc${_2}$O${_3}$ surface. The coalescence of GaN islands on such a rough ScN surface results in incomplete coverage, leaving pores within the GaN nucleation layer.

## Conclusions

In summary, we studied the epitaxial growth of GaN on a $\hbox {Sc}_{{2}}$ $\hbox {O}_{{3}}$(111)/Si(111) buffer layer. The X-ray diffraction (XRD) analysis revealed a cubic and hexagonal GaN phase formation during the growth. A three-fold symmetry of the cubic GaN phase showed two distinct in-plane orientations rotated by 60$^\circ$ to each other, indicating stacking twins. The total emission spectrum of the grown structure consists of three pronounced peaks in the ultraviolet wavelength range. Two narrow peaks belong to GaN near band edge emission of different phase: hexagonal and cubic. A broad peak is attributed to the $\hbox {Sc}_{{2}}$ $\hbox {O}_{{3}}$. The epitaxial GaN films exhibit tensile strain, and micro-strip structures consisting of cubic GaN formed during growth in an N${_2}$ atmosphere was observed. Introducing H${_2}$ into the process suppresses micro-stripe formation, promotes relaxation, and reduces dislocation density in the GaN layer. Two distinct GaN strain states were observed in the epitaxial GaN films separated by an AlGaN interlayers. The upper GaN layer is more relaxed than the layer below. The insertion of AlGaN interlayers reduces both the residual stress and the dislocation density in the GaN films, leading to the successful growth of crack-free GaN in the final stages.

## Methods

**Fig. 12:**
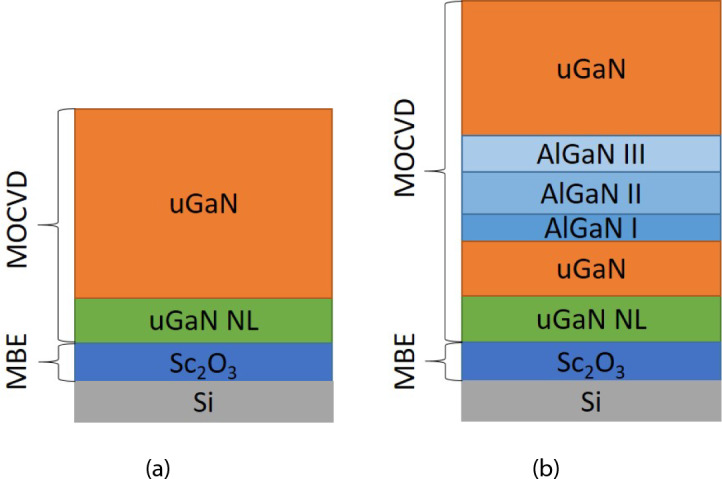
Schematics of GaN structure deposited on $\hbox {Sc}_{{2}}$ $\hbox {O}_{{3}}$(111)/Si(111): (**a**) without (samples No. 1–10), (**b**) with three AlGaN interlayers (sample No. 11).

The Si wafers used in the experiments had a (111) plane orientation. The templates with 40 nm Sc${_2}$O${_3}$(111) buffer layers on Si were prepared in an MBE chamber. These templates were transferred to a closely coupled AIXTRON (3x2) MOVPE reactor for GaN deposition. Trimethylgallium and ammonia were precursors for the gallium and nitrogen sources, respectively, while N${_2}$ or H${_2}$ was chosen as a carrier gas. The trimethylgallium flow was 168 $\upmu \hbox {mol/min}$, while the ammonia flow was 112 mmol/min, resulting in a V/III ratio 664 during high-temperature GaN growth. A total of 11 samples, divided into four groups according to the growth parameters, were considered for this study. The first group of samples No. 1–4 were grown in an N${_2}$ atmosphere at 1050$^{\circ }$C, with varying nitridation times of 300 s, 600 s, 1200 s, and 1800 s. The GaN growth time was constant for those samples, but the optical thickness varied between 360 nm and 460 nm. A nitridation time of 1200 s was selected as optimal for subsequent samples. The different growth time was chosen for the second group of samples No. 5–7. Their total thickness was 430 nm, 310 nm, and 190 nm, respectively. The third group, No. 8–10, differently from the previous two groups, was grown in an H${_2}$ atmosphere. The temperature was 1050$^{\circ }$C, 1070$^{\circ }$C and 1090$^{\circ }$C. Sample No. 11 belongs to the fourth group. This sample, grown in a H${_2}$ atmosphere at 1050$^{\circ }$C, included an additional structure consisting of three AlGaN interlayers with thicknesses of 30 nm, 120 nm, and 60 nm, corresponding to the highest, medium, and lowest Al content, respectively. These three layers were inserted after 130 nm of GaN growth. The total GaN optical thickness for this sample was 570 nm, similar to the third group samples, resulting in a 440 nm thick top GaN layer of sample No. 11. It is important to note that although the GaN growth time was the same for all samples (except for samples No. 6 and 7), the optical thicknesses varied due to differences in surface roughness. Surface defects acted as light-scattering centers, which were not accounted for in the optical thickness evaluation. The schematics of two distinguished uGaN structures grown by MOVPE are presented in Fig. 12.

The growth process started with a low-temperature nucleation layer at 500$^{\circ }$C, followed by high-temperature GaN epitaxy at 1050–1090$\,^{\circ }$C. An $\textit{in situ}$ interferometer, EpiTT from Laytec, using light-emitting diodes with wavelengths of 405 nm, 633 nm, and 950 nm, was employed to monitor the growth rate and surface morphology. Additionally, the total thickness of the GaN structures grown by MOVPE was measured at room temperature using an interferometer from AvaSpec. These thickness values, along with other growth parameters, are summarized in Table 1. The surface and the cross-sectional structure were examined using a scanning electron microscope (SEM). Cathodoluminescence (CL) imaging was performed at room temperature with an acceleration voltage of 4 kV. The electron beam was scanned with a step size of 6.5 nm and a dwell time of 9.7 ms. Atomic force microscopy (AFM) was employed for detailed surface analysis at the atomic scale. The structural analysis was conducted using high-resolution X-ray diffraction (XRD) with a Cu K$\alpha$ radiation source. XRD was employed to identify the phases present during the epitaxy process, assess the lattice alignment of the structure, and determine dislocation densities. The in-plane strain from XRD measurements was evaluated using the following procedure. By fitting the Voigt function, the maximum of the $2\theta$ peak was determined from the symmetric $2\theta -\omega$ scan of the 0004 reflection. The out-of-plane strain $\varepsilon _{zz}$ was calculated using Bragg’s law. This out-of-plane strain was then converted into in-plane strain $\varepsilon _{xx}$ using the equation $\varepsilon _{xx}=[(1-\nu )/2\nu ]\varepsilon _{zz}$, where the Poisson’s ratio, $\nu$, was taken as 0.183^47,60^. Finally, Raman spectroscopy, excited with a 532 nm laser, was used to compare the stress state findings obtained from the XRD measurements.

**Table 1:** Growth parameters for GaN samples.

Group	No.	Atmosphere	Temperature	Nitridation time	Total thickness	Comments
1	1	N_2_	1050	300	440	–
	2	N_2_	1050	600	440	–
	3	N_2_	1050	1200	460	Optimal nitridation time
	4	N_2_	1050	1800	360	The highest amount of cub-GaN
2	5	N_2_	1050	1200	430	–
	6	N_2_	1050	1200	310	–
	7	N_2_	1050	1200	190	–
3	8	H_2_	1050	1200	540	–
	9	H_2_	1070	1200	550	–
	10	H_2_	1090	1200	560	–
4	11	H_2_	1050	1200	780	Includes 3 AlGaN interlayers

## Supplementary Information

Supplementary Information.

## Data Availability

The datasets used and/or analyzed during the current study are available from the corresponding author on reasonable request. Some data on the epitaxy of GaN will be included in a patent at the end of the project (S-MIP-23-42) and are, therefore, not yet publicly available.
