# Supplementary material for: GaN metal-organic vapor phase epitaxy on Sc2O3/Si templates for group III-nitride monolithic integration to Si technology
Source: Sci Rep. 2025 Jul 27;15:27316. doi: 10.1038/s41598-025-12904-9 (PMC12301438; doi:10.1038/s41598-025-12904-9)
Supplement: Supplementary file 1 — Supplementary Information. [file 41598_2025_12904_MOESM1_ESM.pdf]

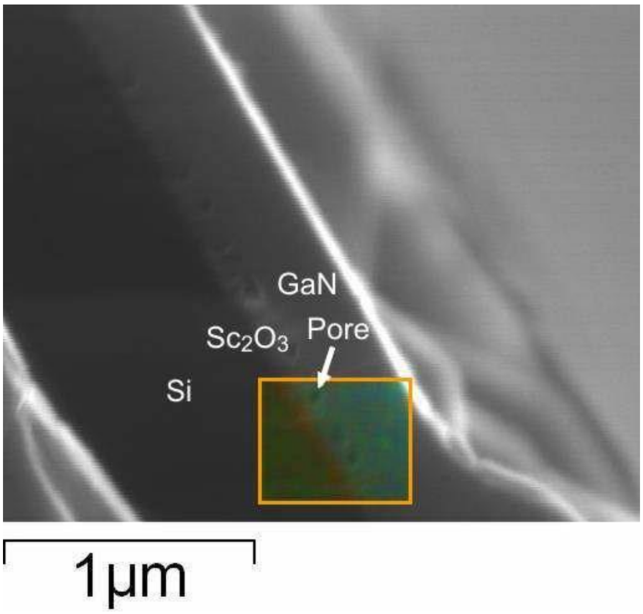

Fig. S1 SEM image and corresponding EDX map.

Fig. S1. SEM image and corresponding energy-dispersive X-ray (EDX) elemental map of the cross-section of GaN grown on Sc_2O_3 (111)/Si(111) in a nitrogen atmosphere (Sample No. 4). The colours represent the distribution of X-ray energies in the 3.5–10 keV range. Red indicates the emission of the Sc Kα line (4 keV), while blue corresponds to the Ga Kα line (9.2 keV). The image confirms that pores were formed within the GaN nucleation layer, located above the Sc_2O_3 buffer layer.

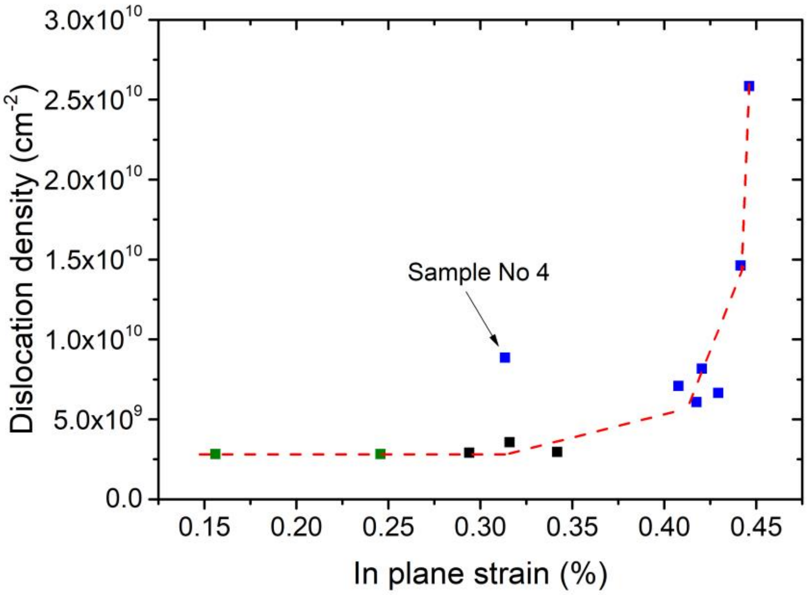

Fig. S2. Dislocation density vs. In-plane strain.

Fig. S2 illustrates the relationship between dislocation density and in-plane strain in GaN layers. The results indicate that dislocation density decreases as the GaN layers undergo relaxation. However, Sample No. 4, which was grown with the longest nitridation time, deviates from this trend, suggesting a different relaxation mechanism.

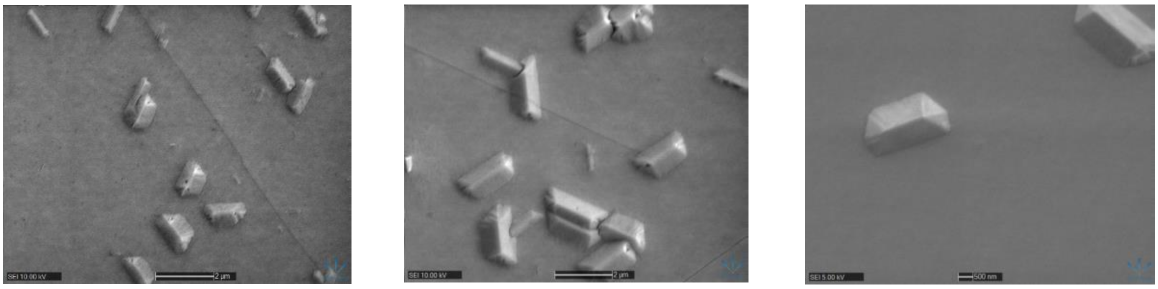

Sample No 7
Optical thickness 190 nm

Sample No 6
Optical thickness 310 nm

Sample No 3
Optical thickness 460 nm

Fig. S3. GaN layers grown at the same nitridation time of 1200 s, but different growth duration times. SEM images (the same magnification).

Fig. S3 shows that stripe-type structures originate from the early stage of GaN growth. The size of stripe-type structures increases with growth time.
